# Supplementary material for: Synergistic RAS-MAPK and AKT Activation in MYC-Driven Tumors via Adjacent PVT1 Rearrangements
Source: bioRxiv. 2025 Feb 22:2025.02.17.638454. Preprint. [Version 1] doi: 10.1101/2025.02.17.638454 (PMC11870553; doi:10.1101/2025.02.17.638454)
Supplement: Supplement 2 [file NIHPP2025.02.17.638454v1-supplement-2.pdf]

(which was not certified by peer review) is the author/funder, who has granted bioRxiv a license to display the preprint in perpetuity. It is m
available under aCC-BY 4.0 International license.

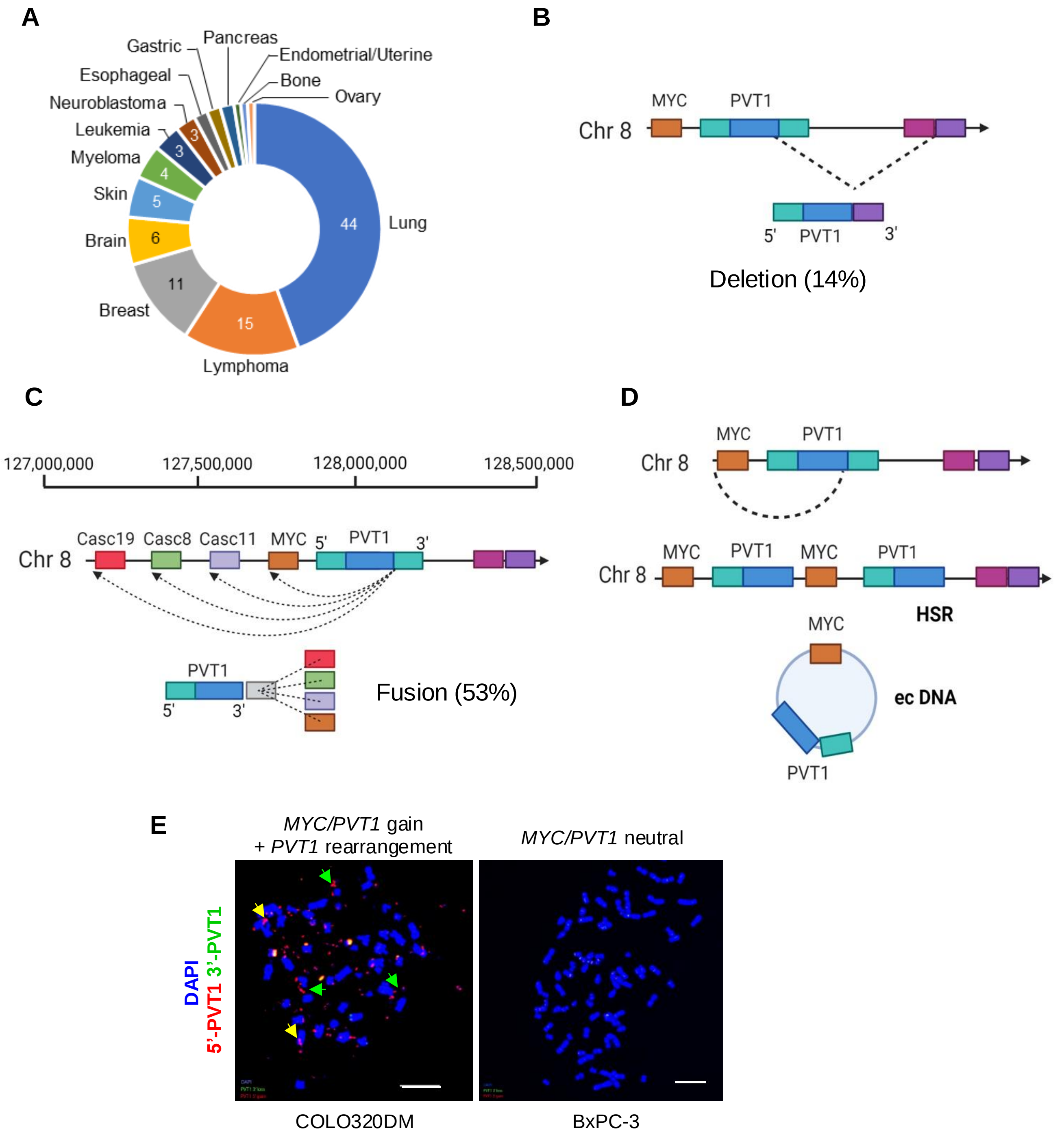

Supplementary Figure 1: PVT1 fusions in cancers (A) Donut plot showing the distribution of PVT1 fusion-positive cancers in the CCLE dataset. The slice for each cancer type is proportional to the number of cell lines for that cancer type. A total of 115 cell lines were positive for PVT1 fusion (n=115). (B) 14% of intrachromosomal PVT1 fusions harbor deletion of the PVT1 gene. (C, D) 53% of intrachromosomal rearrangements fuse 5'-PVT1 to a gene partner on 8q24 located upstream of PVT1, such as CASC11, CASC8, or MYC, suggesting that such fusions may generate extrachromosomal DNA (ecDNA) and/or double minute chromosomes (HSRs). (E) Representative dual probe (5'-PVT1/3'PVT1) FISH images of COLO320DM (MYC/PVT1 gain + PVT1 rearrangement) and BxPC-3 (MYC/PVT1 neutral) cell lines. COLO320DM showed 5' enrichment of PVT1 on ecDNA and HSR, as shown by the green and yellow arrows, respectively. BxPC-3 cells were used as control. Scale bars, 10 µm.

(which was not certified by peer review) is the author/funder, who has granted bioRxiv a license to display the preprint in perpetuity. It is m
available under aCC-BY 4.0 International license.

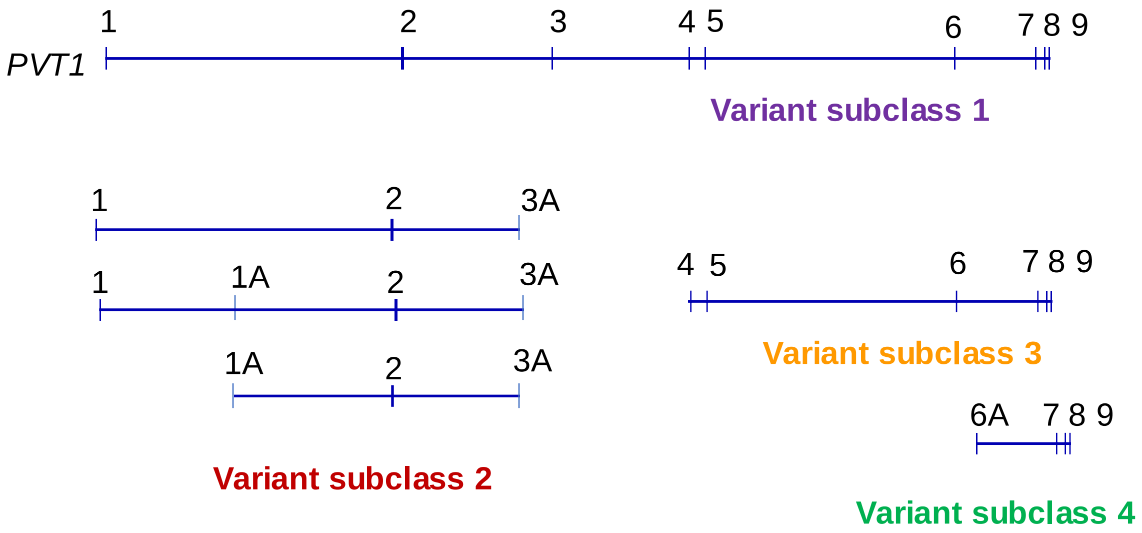

Supplementary Figure 2: Broad subcategorization of 183 PVT1 transcript variants (ENSG00000249859) The primary PVT1 transcript contains nine exons (1-9). 183 PVT1 transcript variants were identified. These variants were broadly categorized into four subclasses: Variant subclass 1 (18 variants): Contains full-length PVT1. Variant subclass 2 (67 variants): Contains exons 1, 2, and 3A, and similar iterations spanning 5'-PVT1. Variant subclass 3 (86 variants): Contains exons 4A-9 (and their different sub-iterations). Variant subclass 4 (22 variants): Contains exons 6A-9. (and their different sub-iterations). The distribution of variants across subclasses is shown in Supplementary Table 1.

(which was not certified by peer review) is the author/funder, who has granted bioRxiv a license to display the preprint in perpetuity. It is m
available under aCC-BY 4.0 International license.

A **Sequence of *PVT1* exon 2**

5'GCCTGATCTTTTGGCCAGAAGGAGATTAAAAAGATGCCCCTCAAGATGGCTGTGCCTGTCAGCTGCATGGAG
CTTCGTTCAAGTATTTTCTGAGCCTGATGGATTTACAGTGATCTTCAGTGGTCTGGGGAATAACGCTGGTGGAA
CCATGCACTGGAATGACACACGCCCGGCACATTTCAGGATACTAAAAGTGGTTTTAAGGGAGGCTGTGGCTGA
ATGCCTCATGGATTCTTACAGCTTGGATGTCCATGGGGGACGAAGGACTGCAGCTGGC**TGA**GAGGGTTGAGA
TCTCTGTTTACTTAGATCTCTGCCAACTTCCTTTGGGTCTCCCTATGGAATGTAAGACCCCGACTCTTCCTGGTG
AAGCATCTG**ATG**CACGTTCCATCCGGCGCTCAGCTGGGCTTGAG-3'

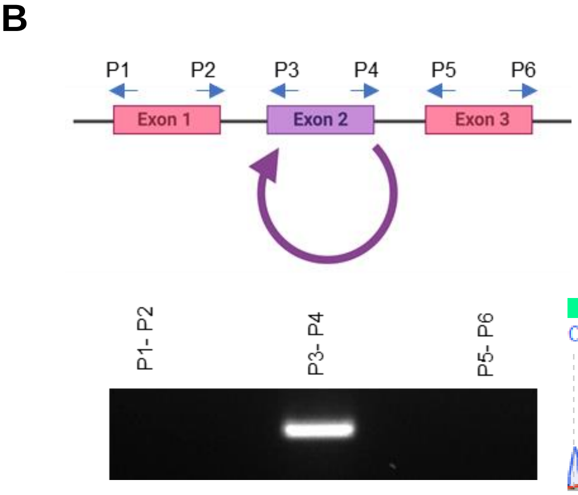

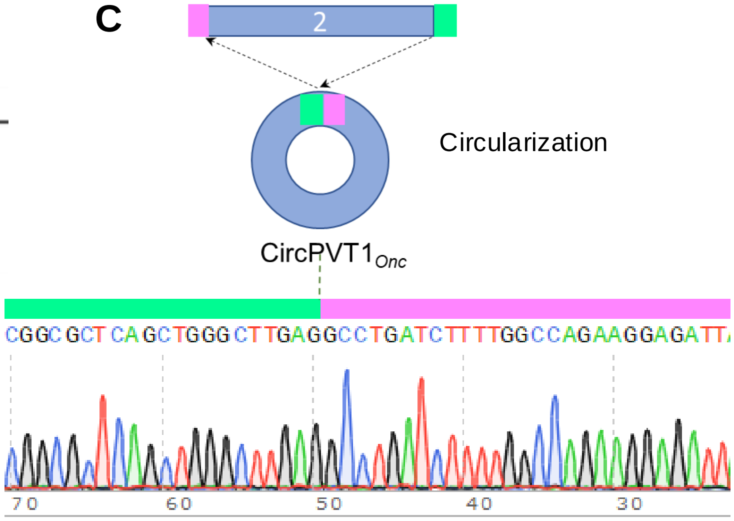

Confirmation of circularization by sequencing

D **Sequence of circularized *PVT1* exon 2**

TGCCAACTTCCTTTGGGTCTCCCTATGGAATGTAAGACCCCGACTCTTCCTGGTGAAGCATCTG**ATG**CACGTTC
CATCCGGCGCTCAGCTGGGCTTGAGGCCTGATCTTTTGGCCAGAAGGAGATTAAAAAGATGCCCCTCAAGATG
GCTGTGCCTGTCAGCTGCATGGAGCTTCGTTCAAGTATTTTCTGAGCCTGATGGATTTACAGTGATCTTCAGTG
GTCTGGGGAATAACGCTGGTGGAACCATGCACTGGAATGACACACGCCCGGCACATTTCAGGATACTAAAAGT
GGTTTTAAGGGAGGCTGTGGCTGAATGCCTCATGGATTCTTACAGCTTGGATGTCCATGGGGGACGAAGGACT
GCAGCTGGC**TGA**GAGGGTTGAGATCTCTGTTTACTTAGATCTC

PVT1 exon2 circular primer sequence 288bp
Forward: 5' TGC CAA CTT CCT TTG GGT CTC 3'
Reverse: 5' AGT ATC CTG AAA TGT GCC GGG 3'

Supplementary Figure 3: PVT1 exon 2 forms Circular RNA (A) Nucleotide sequence of PVT1 exon 2. The 5' and 3' ends of exon 2 are highlighted by pink and green color, respectively. The translation start and stop codons are highlighted. (B) Schematic representation of divergent primer pairs (P1/P2, P3/P4, and P5/P6) for respective exons to identify circularization. Agarose gel electrophoresis of amplified product from cDNA (prepared by random hexamer oligonucleotides) using the described primer pairs. (C) Schematics of exon 2 circularization and confirmation of junction sequence (joining of 5'/3' ends) by sequencing of amplified product (P3/P4 primer pair). (D) Nucleotide sequence of circularized exon 2 and resulting new ORF is underlined with start and stop codon highlighted in red and blue color, respectively.

(which was not certified by peer review) is the author/funder, who has granted bioRxiv a license to display the preprint in perpetuity. It is m
available under aCC-BY 4.0 International license.

A

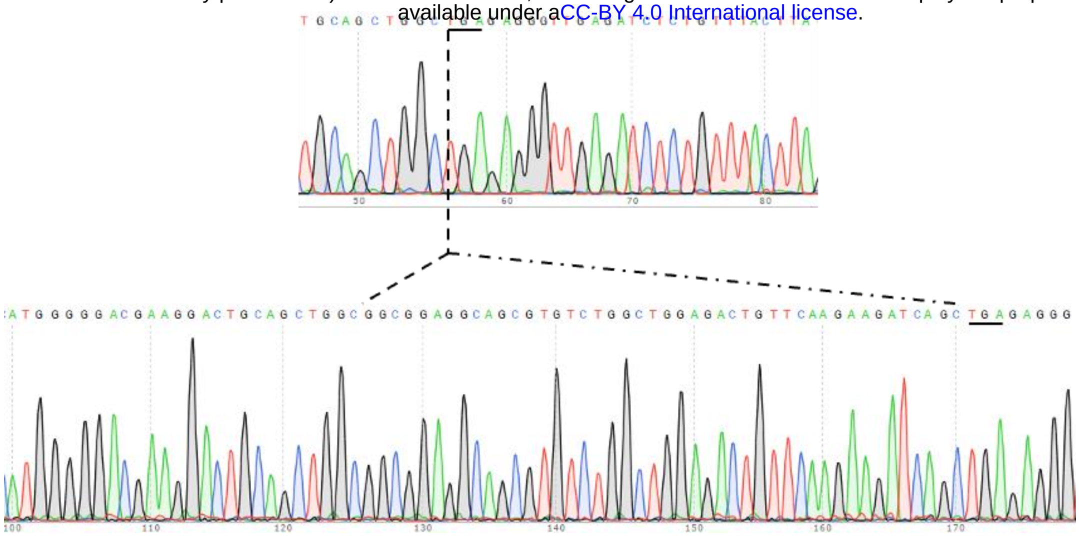

B

Control (Untagged FFX) **FFX-HiBiT**

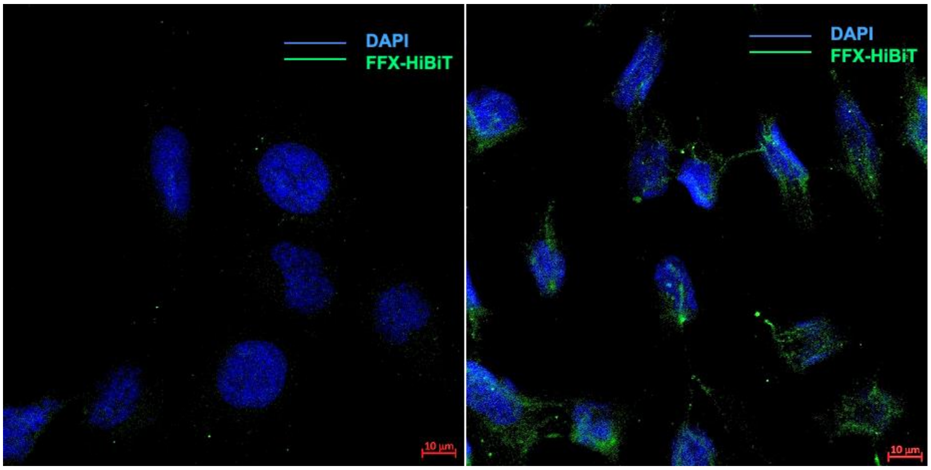

Supplementary Figure 4: Endogenous tagging of FFX with HiBiT. (A) Sequence chromatograms of HiBiT sequence fused to endogenous FFX (immediately upstream of native stop codon to generate C-terminal protein fusion) using CRISPR/Cas9 knock-in. The nucleotide sequences of the HiBiT tag are also shown. (B) Immunofluorescent detection of HiBiT-tagged FFX in CRISPR-edited cell clones using the anti-HiBiT monoclonal antibody (green) and DAPI (blue). Scale bars, 10 µm.

(which was not certified by peer review) is the author/funder, who has granted bioRxiv a license to display the preprint in perpetuity. It is m
available under aCC-BY 4.0 International license.

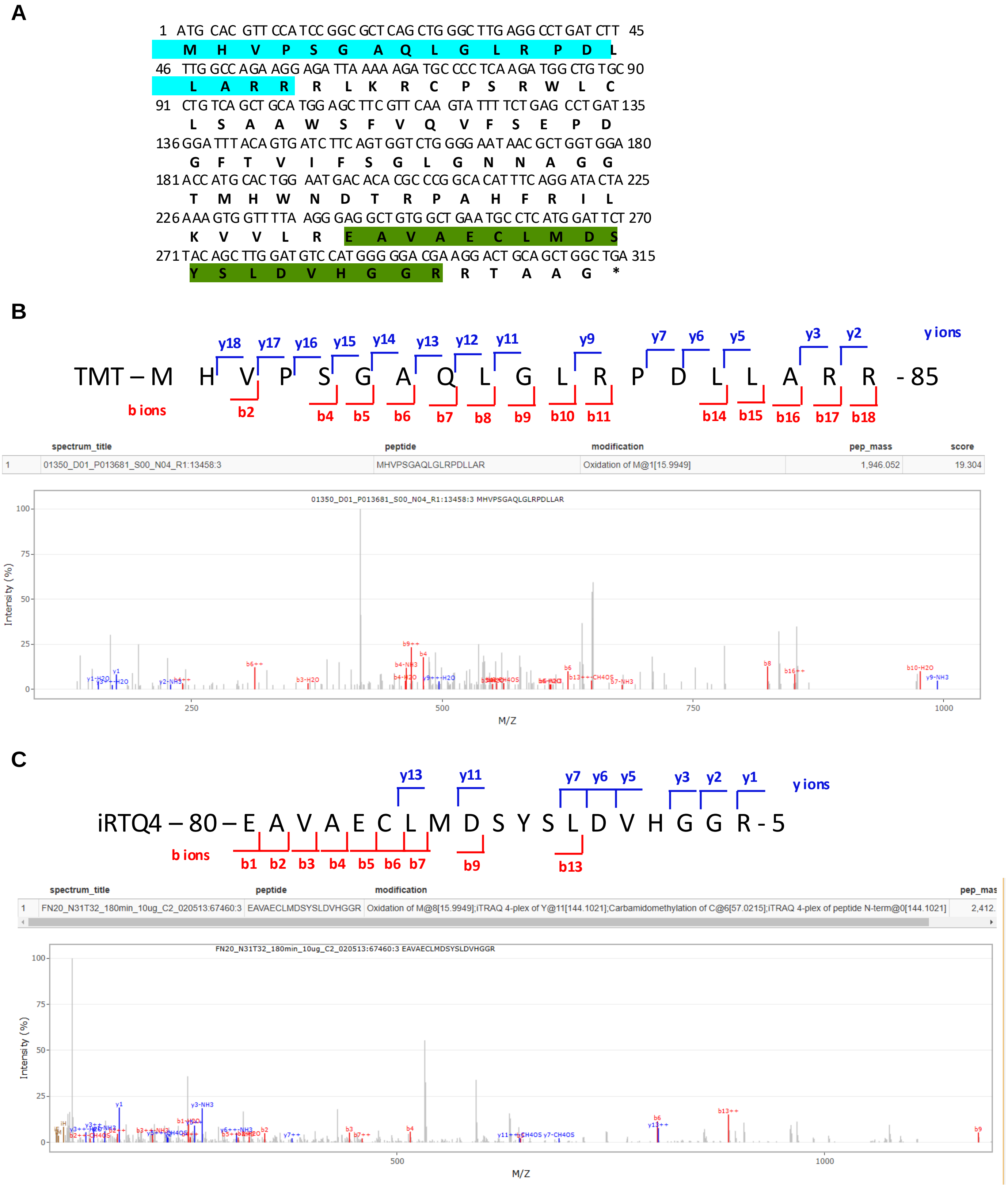

Supplementary Figure 5: Identification of FFX peptide sequence in human proteomic databases. **(A)** FFX sequence with the highlighted regions corresponding to the peptides identified in the human proteomics database. **(B, C)** Representative Mass spectra image of FFX peptides MHVPSGAQLGRPDLLAR and EAVAECLMDSYSLDVHGGR identified from the human proteomics database. The y-axis represents relative ion abundance, while the x-axis represents the m/z ratio.

(which was not certified by peer review) is the author/funder, who has granted bioRxiv a license to display the preprint in perpetuity. It is m
available under aCC-BY 4.0 International license.

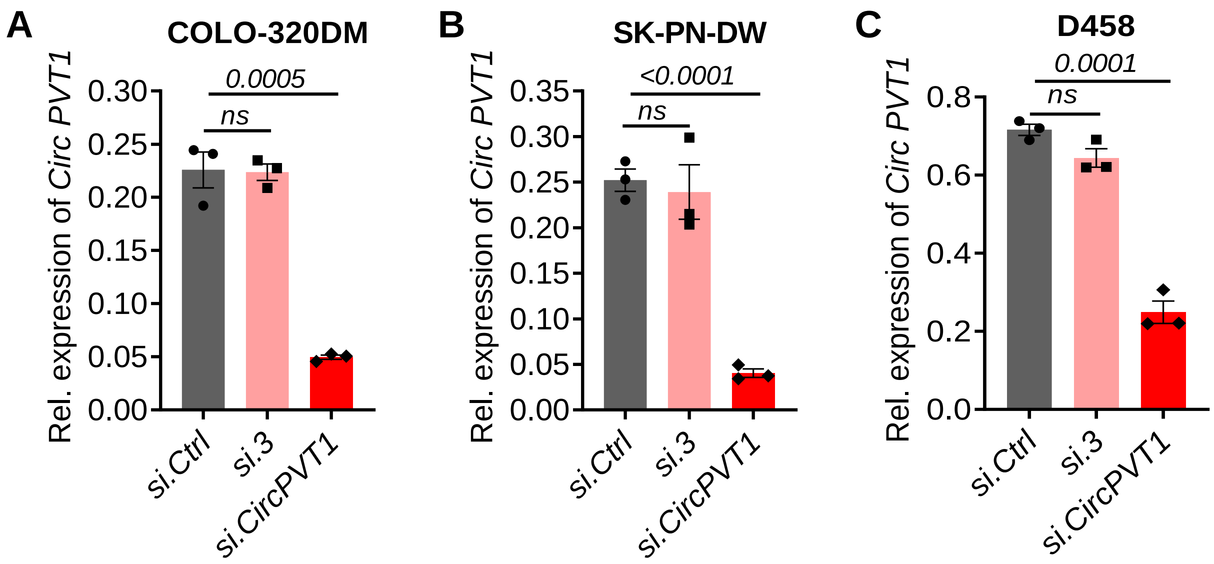

Supplementary Figure 6: siRNA mediated knockdown of CircPVT1. qRT-PCR of CircPVT1 transcript from (A) COLO-320DM, (B) SK-PN-DW and (C) D458 cells after transfection with si.Ctrl (control siRNA), si.3 (siRNA targeting linear PVT1), and si.CircPVT1 (siRNA targeting CircPVT1). (n=3, P values were determined by unpaired t test.)

(which was not certified by peer review) is the author/funder, who has granted bioRxiv a license to display the preprint in perpetuity. It is m
available under aCC-BY 4.0 International license.

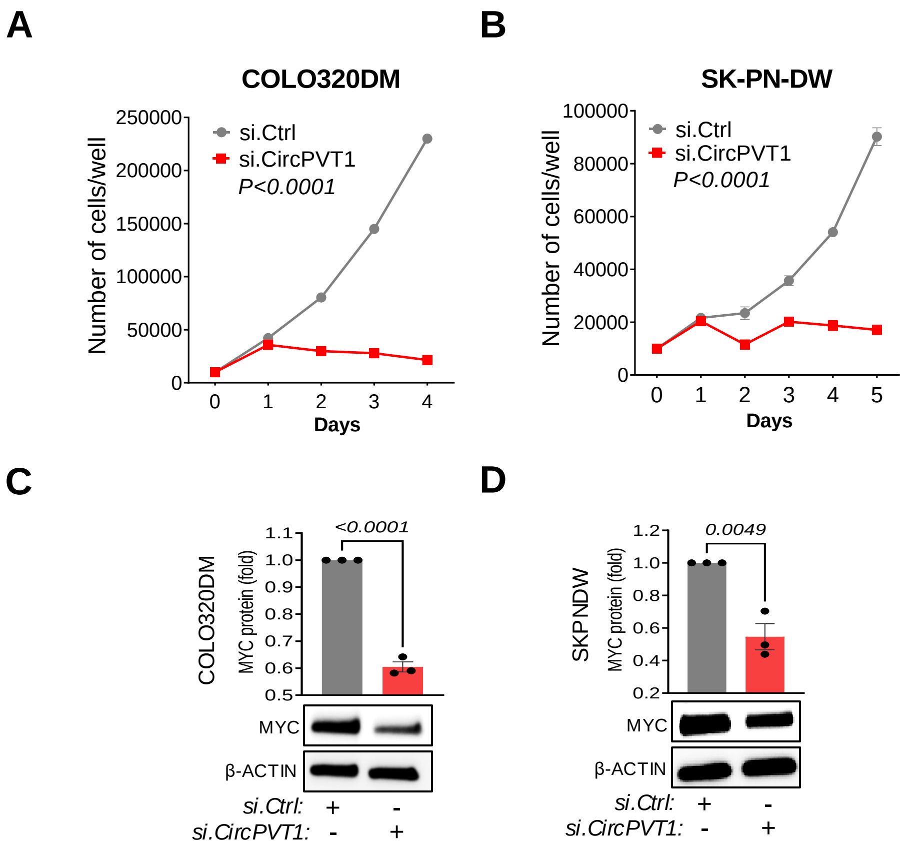

Supplementary Figure 7: Cell proliferation assay of COLO320DM (A) and SK-PN-DW(B) after transfection with si.CircPVT1 or si.Ctrl (n=3, P value by two-way ANOVA). (C-D) Western blot analysis of MYC expression in COLO320DM and SK-PN-DW transfected with si.Ctrl or si.CircPVT1. β ACTIN was used as loading control (n=3, P value by unpaired t test).

(which was not certified by peer review) is the author/funder, who has granted bioRxiv a license to display the preprint in perpetuity. It is m
available under aCC-BY 4.0 International license.

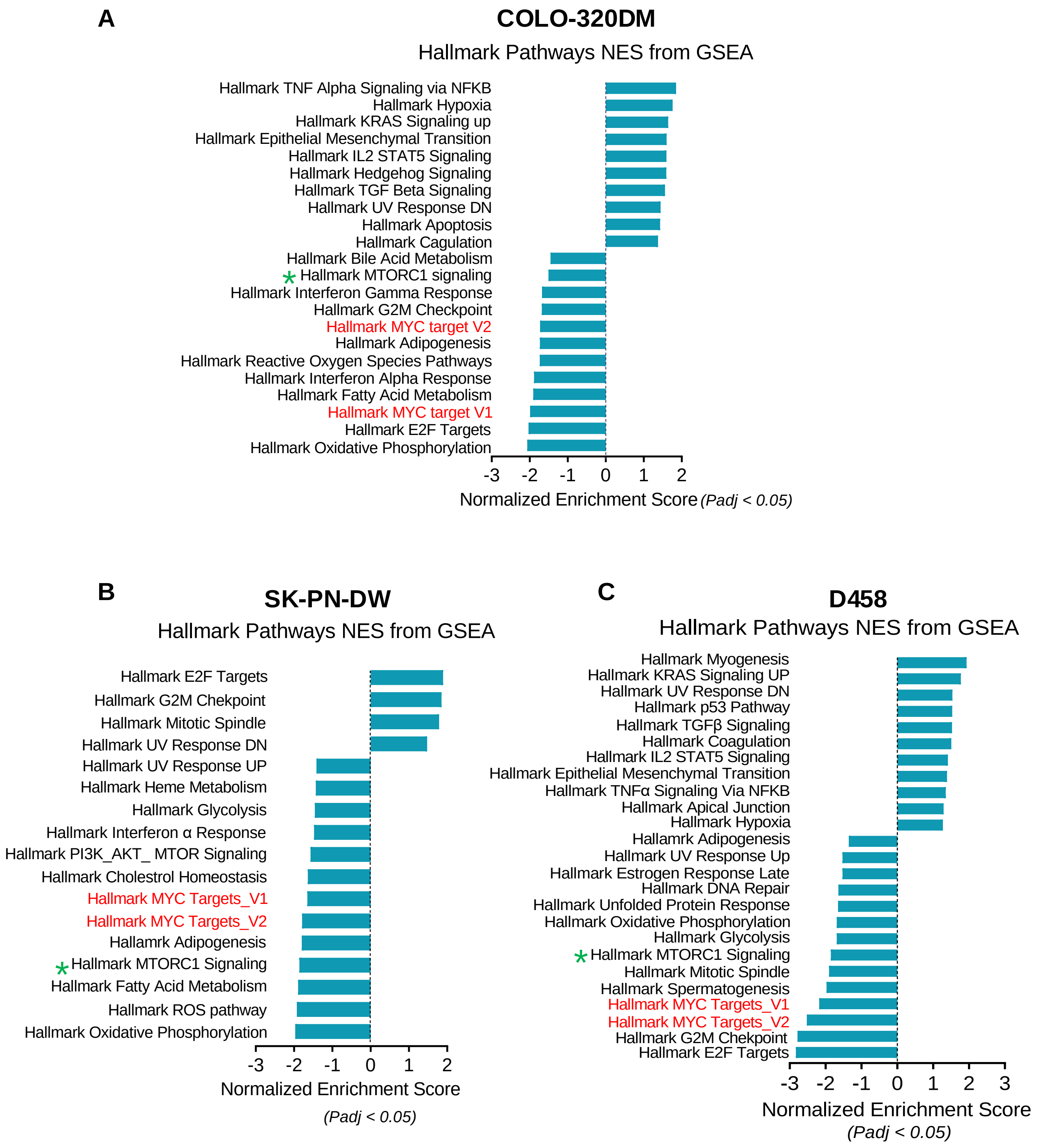

Supplementary Figure 8: Global hallmark pathway analysis of RNA Seq data from si.CircPVT1 vs si.Ctrl treated cells. Gene set enrichment analysis (GSEA) of differentially expressed protein pathways in (A) COLO-320DM, (B) SK-PN-DW, and (C) D458 cells after knocking down CircPVT1 (n=3). The X and Y axes represent normalized enrichment scores (NES) and the total list of GSEA hallmark upregulated and downregulated categories, with significantly enriched terms (p-adjust < 0.05). The hallmark MYC targets V1 and V2 (in red) and MTORC1 (in green asterisk) are highlighted

(which was not certified by peer review) is the author/funder, who has granted bioRxiv a license to display the preprint in perpetuity. It is m
available under aCC-BY 4.0 International license.

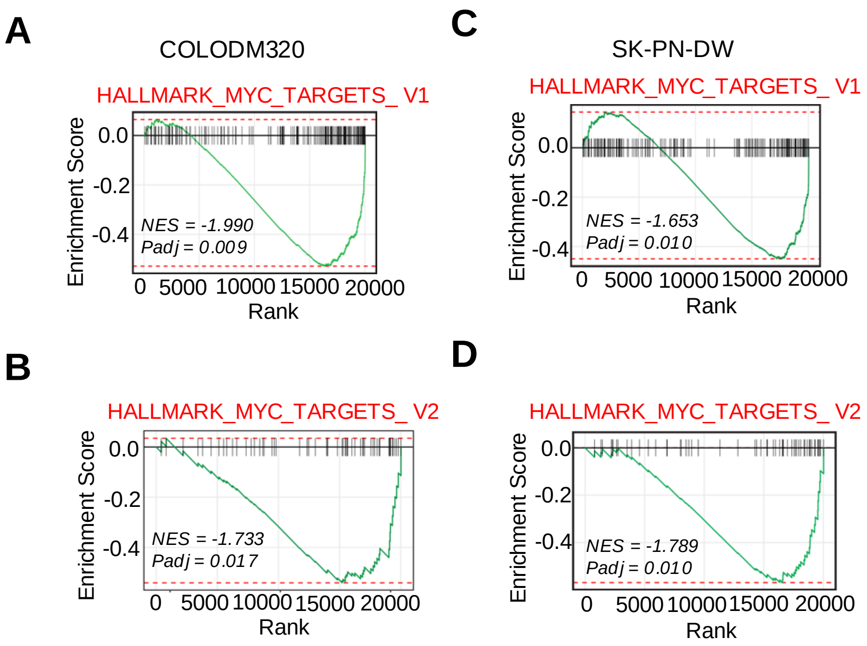

Supplementary Figure 9: Gene set enrichment analysis scores (GSEA) analysis for MYC targets genes in COLODM320 (A,B) and SK-PN-DW (C,D) treated with si.CircPVT1. si.Ctrl was used as control. n=3, for each treatment condition.

(which was not certified by peer review) is the author/funder, who has granted bioRxiv a license to display the preprint in perpetuity. It is m
available under aCC-BY 4.0 International license.

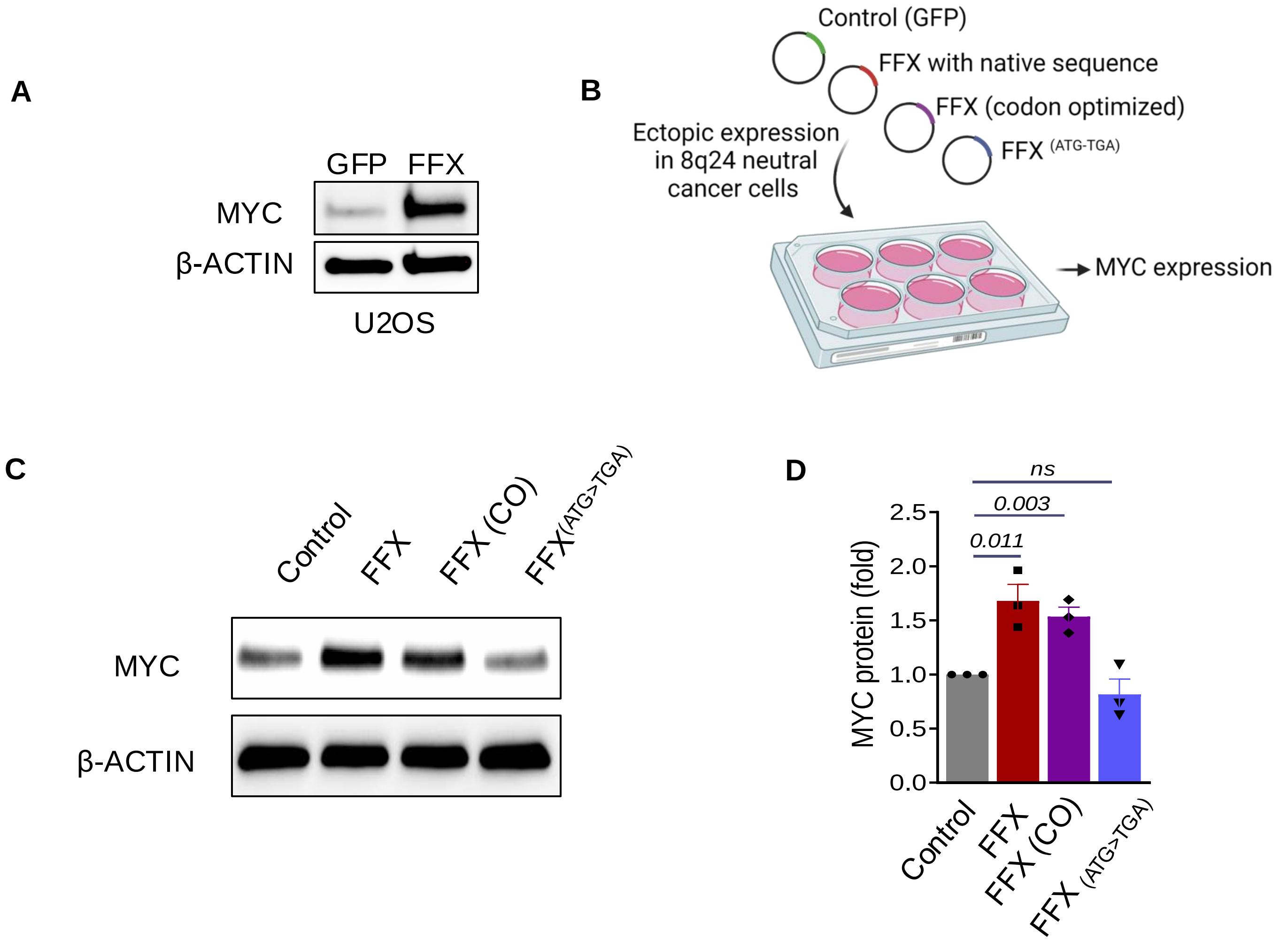

Supplementary Figure 10: FFX protein augments MYC expression **(A)** Western blot analysis of MYC expression in U2OS cells ectopically transduced with constructs expressing GFP (control) or FFX. **(B)** Schematic of assay for MYC expression in U2OS cells ectopically transduced with constructs expressing GFP (control), FFX (with native sequence), codon optimized FFX (FFX(CO)), or mutant FFX in which the start codon ATG is mutated to TGA ($FFX^{ATG>TGA}$). **(C&D)** Western blot analysis and quantification of MYC expression in U2OS cells transduced with GFP (control), FFX, FFX(CO), and FFX $^{ATG>TGA}$. (n=3, p value by unpaired t test).

(which was not certified by peer review) is the author/funder, who has granted bioRxiv a license to display the preprint in perpetuity. It is m
available under aCC-BY 4.0 International license.

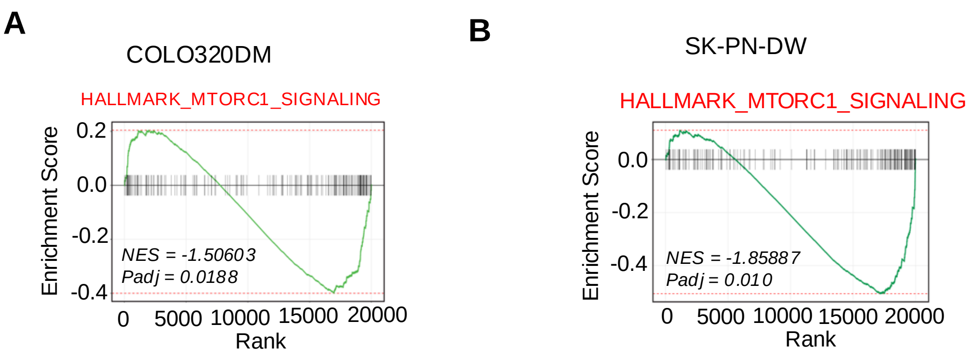

Supplementary Figure 11: Gene set enrichment analysis scores (GSEA) analysis of genes involved in MTORC1 signaling in COLO320DM (A) and SK-PN-DW (B) treated with si.CircPVT1. si.Ctrl was used as control. n=3, for each treatment condition.

(which was not certified by peer review) is the author/funder, who has granted bioRxiv a license to display the preprint in perpetuity. It is m
available under aCC-BY 4.0 International license.

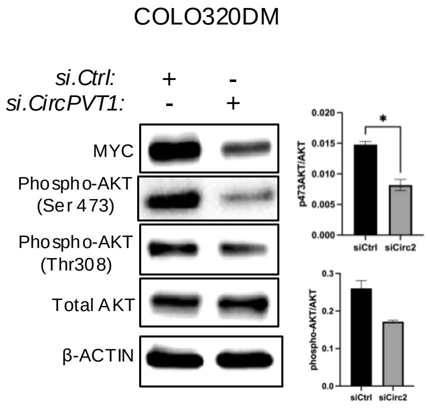

Supplementary Figure 12: Western blot and quantitative analysis of MYC, phospho-AKT (Ser 473 and Thr308) , total AKT expression in D458 cells transduced with si.CircPVT1 and si.Ctrl. β ACTIN was used as a control. (p-values obtained by unpaired t-test, n=3, for each treatment condition)

(which was not certified by peer review) is the author/funder, who has granted bioRxiv a license to display the preprint in perpetuity. It is m
available under aCC-BY 4.0 International license.

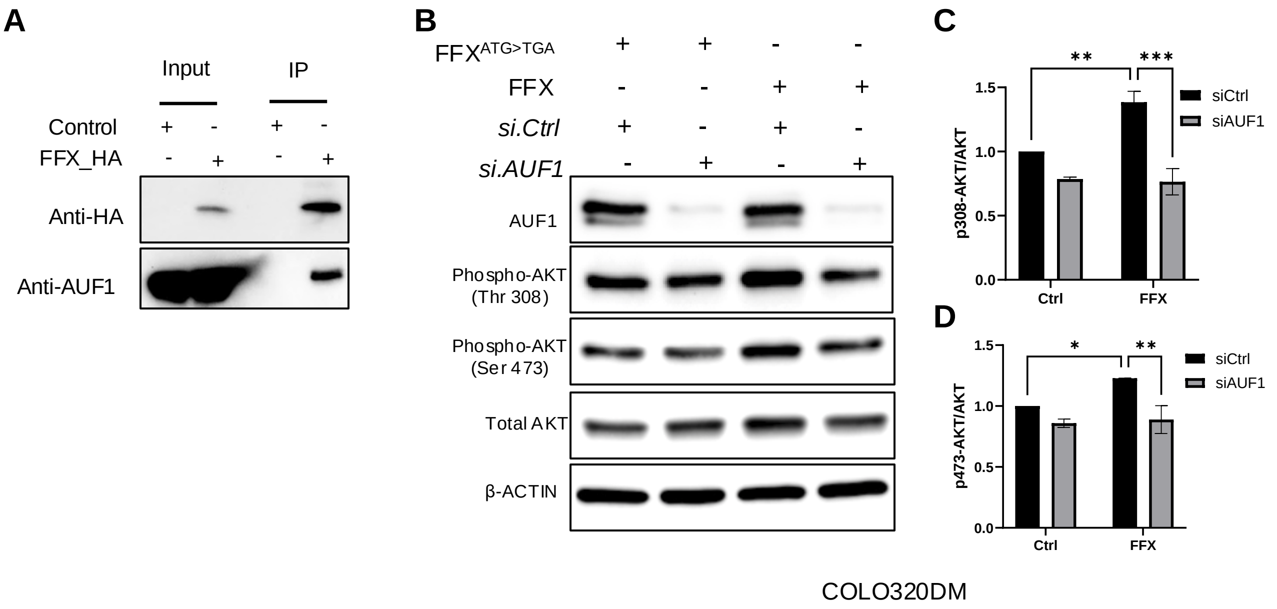

Supplementary Figure 13: A. Western blot analysis of Co-IP experiments from U2OS transfected with FFX (control) or HA tagged FFX (FFX_HA) with indicated antibodies**. B.** Western blot analysis of AUF1, phospho-AKT (Thr308 and Ser 473) , total AKT expression in COLO320DM cells transduced with $FFX^{ATG>TGA}$ (Control) and FFX and treated with si.AUF1 and si.Ctrl. β ACTIN was used as a control. (**C,D**) Quantitative analysis of phospho-AKT (Thr308 and Ser 473) expression in COLO320DM cells transduced with $FFX^{ATG>TGA}$ (Control) and FFX and treated with si.AUF1 and si.Ctrl (p-values obtained by unpaired t-test, n=3, for each treatment condition).

[illegible]
(which was not certified by peer review) is the author/funder, who has granted bioRxiv a license to display the preprint in perpetuity. It is m
available under aCC-BY 4.0 International license.

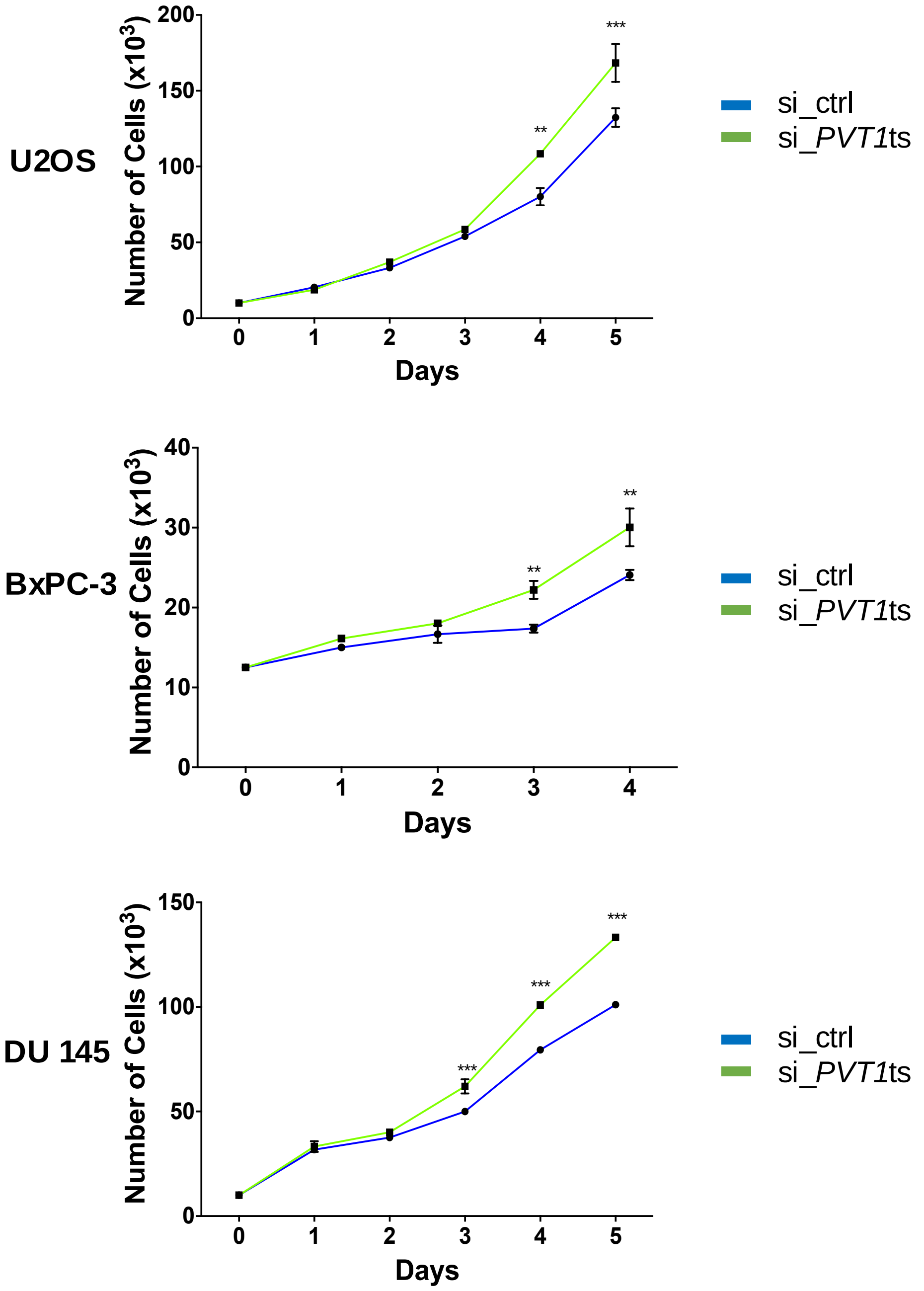

Supplementary Figure 14: Analysis of Cell Proliferation in MYC/PVT1 Neutral Cell Lines (U2OS, BxPC-3, DU-145) following Transfection with si_PVT1ts or si_ctrl: Each experiment was performed in triplicate (n=3), and statistical significance was determined using two-way ANOVA (P values indicated)

(which was not certified by peer review) is the author/funder, who has granted bioRxiv a license to display the preprint in perpetuity. It is m
available under aCC-BY 4.0 International license.

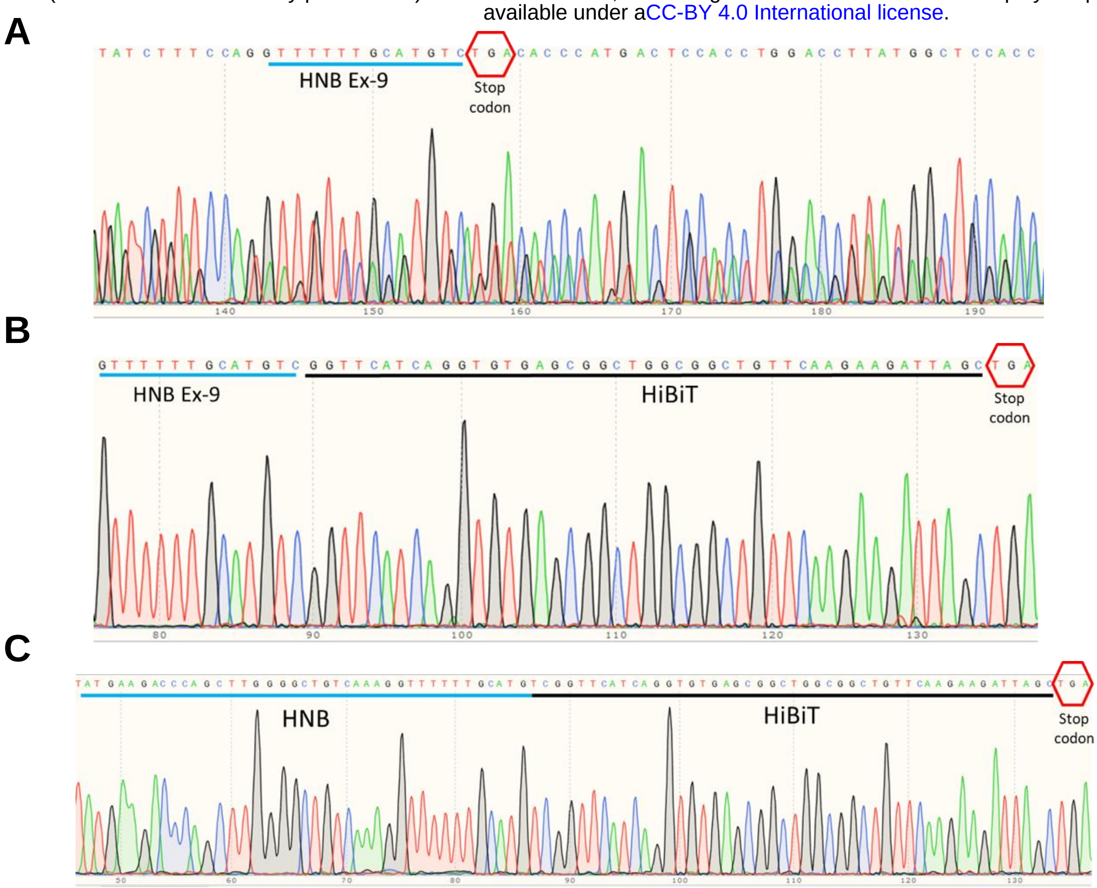

Supplementary Figure 15: CRISPR/Cas9 mediated integration of HiBiT tag at HNB's C-terminus in U2OS cells: Genomic sequence chromatograms comparing native HNB (untagged, **A**) and HiBiT-tagged HNB (**B**). The chromatograms display bioluminescent-negative control cells (A) and bioluminescent-positive HiBiT-tagged U2OS cells (B), confirming the precise knock-in of the HiBiT tag before the stop codon in PVT1 Exon9 of HNB. (C) Sequence chromatograms of HNB-HiBiT cDNA from the HiBiT-positive U2OS cell line, verifying the expression of HNB-HiBiT mRNA

(which was not certified by peer review) is the author/funder, who has granted bioRxiv a license to display the preprint in perpetuity. It is m
available under aCC-BY 4.0 International license.

1 ATG AAG ACC CAG CTT GGG GCT GCT AAA GGT TTT TTG CAT GTC TGA 45
M K T Q L G A V K G F L H V *

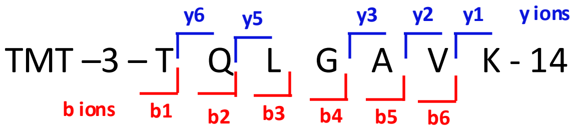

	spectrum_title	peptide	modification	pep_mass	score
1	SecondInstrument_Sample3_121115_Fr10:11220:2	TQLGAVK	TMT 10-plex of K@7[229.1629];TMT 10-plex of peptide N-term@0[229.1629]	1,173.749	23.167

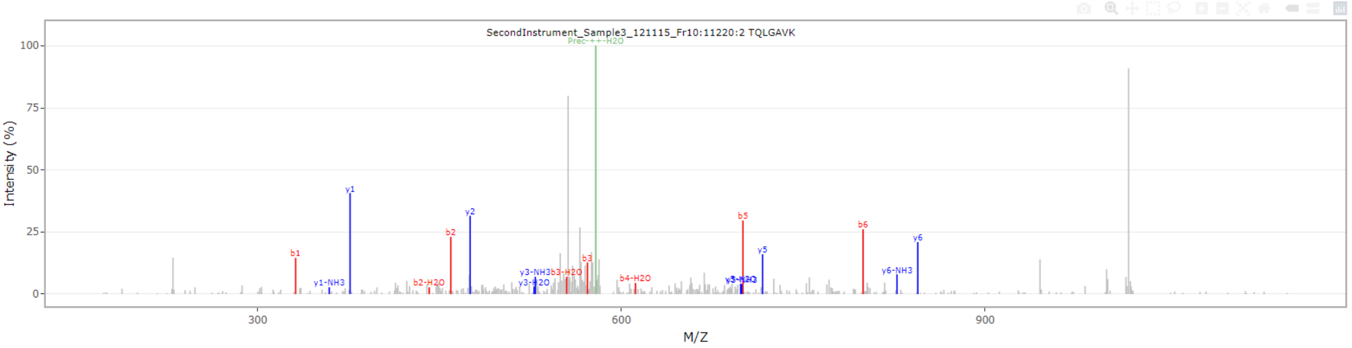

Supplementary Figure 16: Identification of HNB peptide sequence in human proteomic databases. **(A)** HNB peptide sequence with the highlighted region corresponding to the peptide identified in the human proteomic database. **(B)** Representative MS/MS spectra image of HNB peptide TQLGAVK identified from GTEx_32_Tissues_Proteome_PXD016999. The y-axis represents relative ion abundance, while the x-axis represents the m/z ratio.

(which was not certified by peer review) is the author/funder, who has granted bioRxiv a license to display the preprint in perpetuity. It is m
available under aCC-BY 4.0 International license.

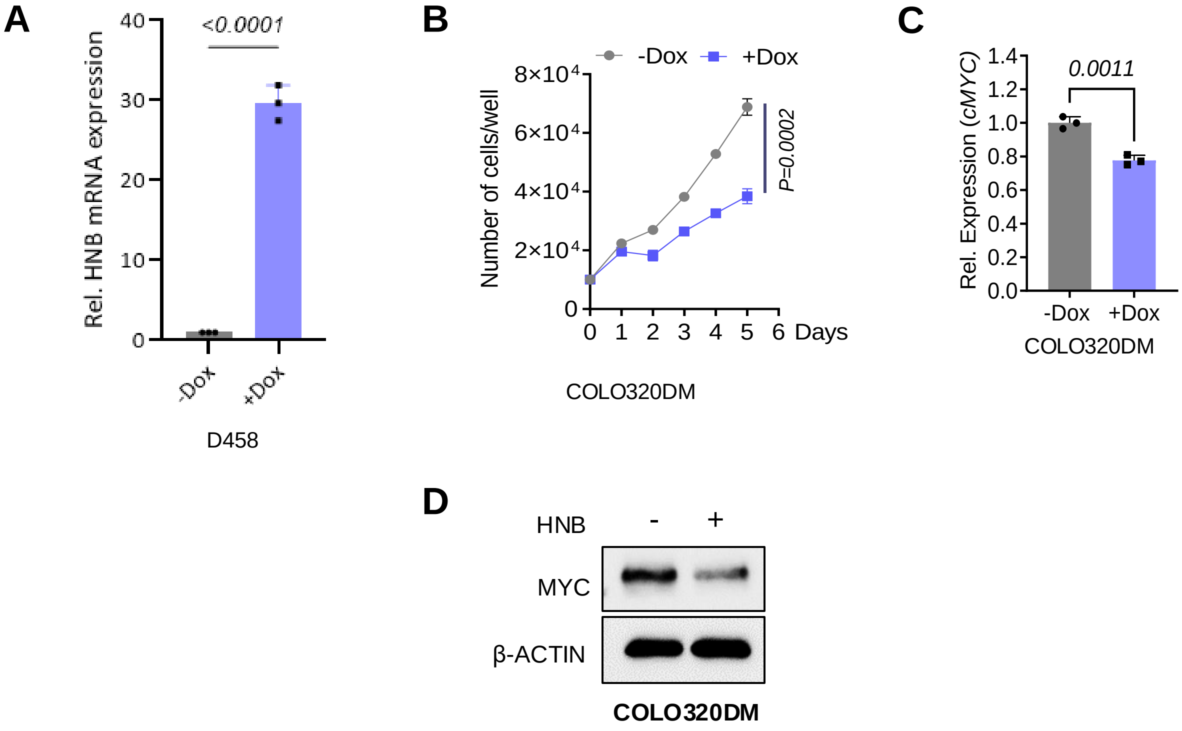

Supplementary Figure 17: Regulation of MYC Expression by HNB: (A) Quantification of HNB transcript levels in D458 cells upon induction (+Dox), as determined by q-RT-PCR. (B) Proliferation assay of COLO320DM cells following HNB induction (-/+ Dox). (C) q-RT-PCR analysis of MYC transcript levels, with or without induction of HNB (-/+ Dox) (n=3, P values obtained through unpaired t-test). (D) Western blot analysis of MYC protein in COLO320DM cells transduced with inducible HNB transgene (-/+ Dox); β-ACTIN serves as the loading control.

(which was not certified by peer review) is the author/funder, who has granted bioRxiv a license to display the preprint in perpetuity. It is m
available under aCC-BY 4.0 International license.

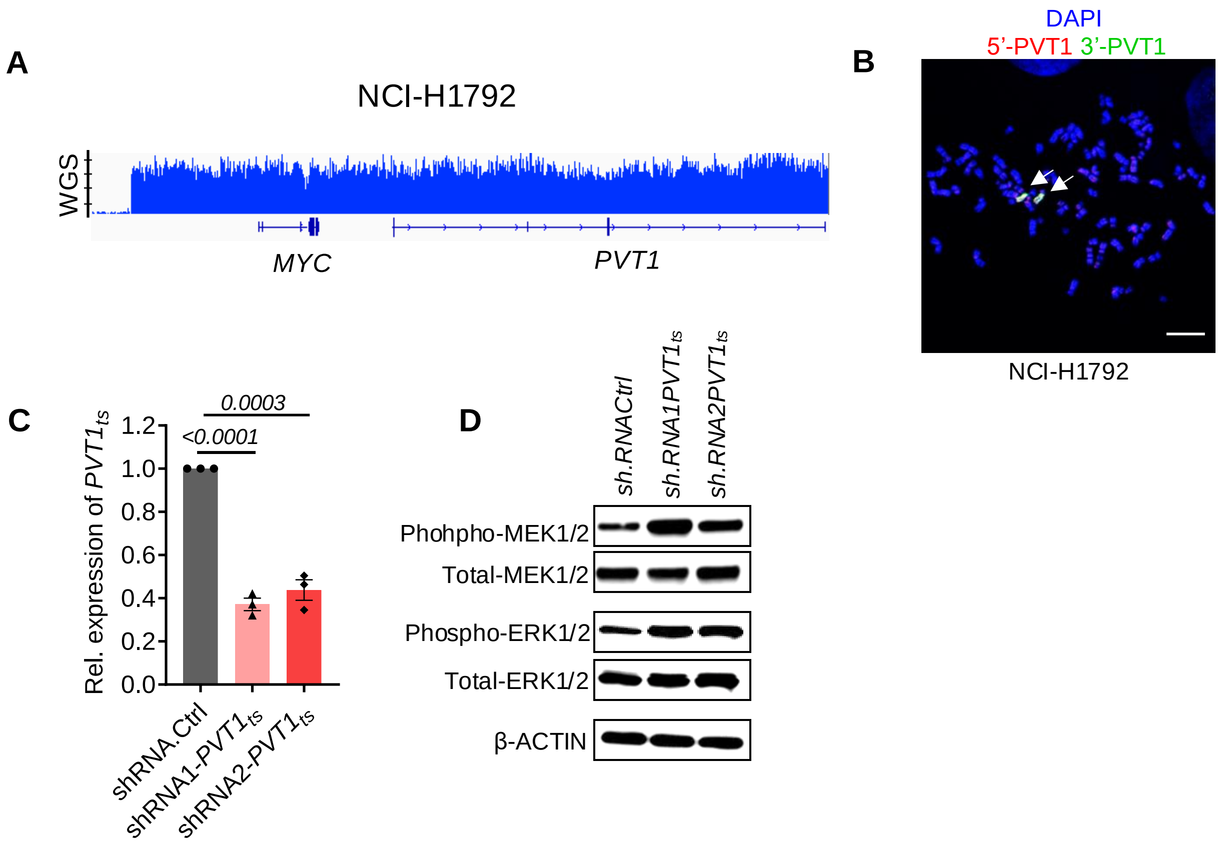

Supplementary Figure 18: Loss of HNB activates RAS/MAPK pathway and stabilizes MYC in cell line with *MYC/PVT1* gain only *(*NCI-H1792) (A) Whole-Genome Sequencing (WGS) coverage across the MYC-PVT1 locus in NCI-H1792 cells **(B)** Representative Fluorescence In Situ Hybridization (FISH) using dual probes (*5'-PVT1*/*3'PVT1)* in NCI-H1792, revealing the intact amplified MYC/PVT1 locus (indicated by arrows). Scale bars 10 µm **(C)** qRT-PCR analysis of $PVT1_{ts}$ transcript levels in NCI-H1792 cells transduced with sh.Cntrl, shRNA1-$PVT1_{ts}$, and shRNA2-$PVT1_{ts}$. **(D)** Western blot assessment of phosphorylated MEK1/2, total MEK1/2, phosphorylated ERK1/2, and total ERK1/2 in NCI-H1792 cells transduced as in (C), with β-ACTIN serving as the loading control.

(which was not certified by peer review) is the author/funder, who has granted bioRxiv a license to display the preprint in perpetuity. It is m
available under aCC-BY 4.0 International license.

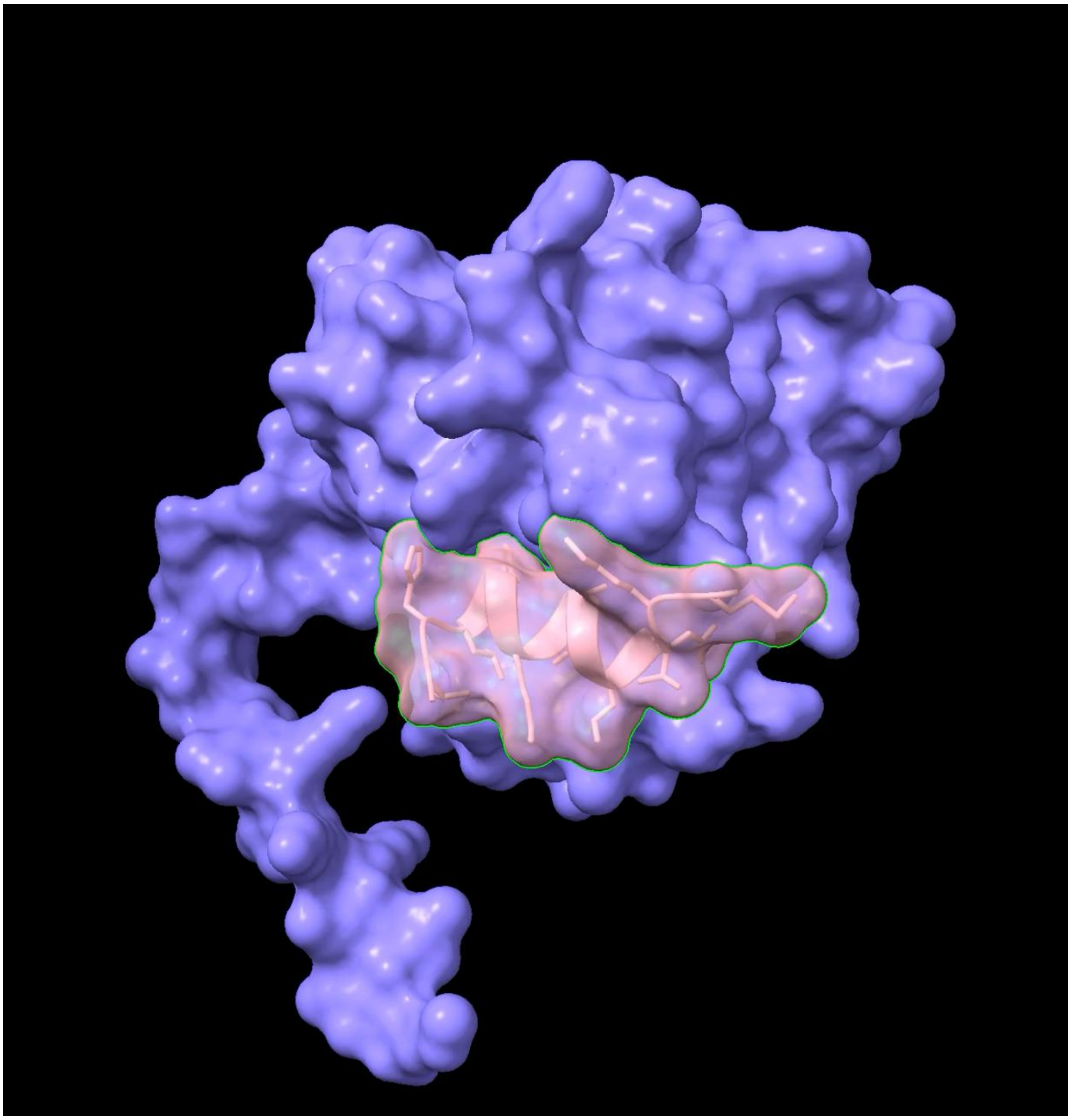

Supplementary Figure 19: Movie showing predicted model of KRAS and HNB derived from AlphaFold 3. KRAS is represented by the purple space-filling model, while HNB is represented by the salmon ribbon and transparent space-filling models.

(which was not certified by peer review) is the author/funder, who has granted bioRxiv a license to display the preprint in perpetuity. It is m
available under aCC-BY 4.0 International license.

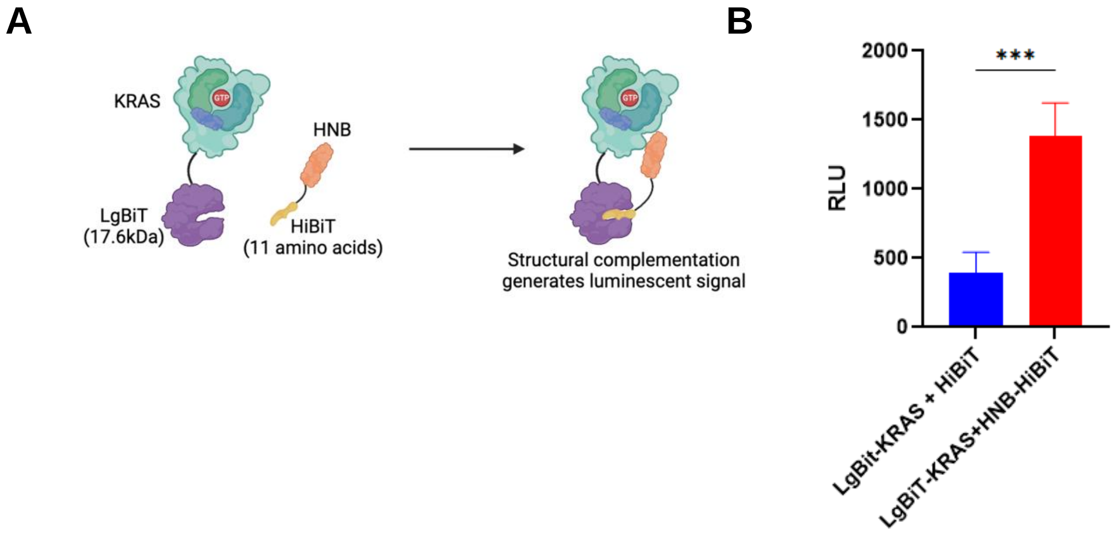

Supplementary Figure 20: Analysis of KRAS and HNB Interaction: (A) Illustration of the NanoBiT protein-protein interaction assay highlighting the specific interaction between LgBiT-tagged KRAS and HiBiT-tagged HNB expressed in HEK293T cells. The complementation between LgBiT and HiBiT, resulting from this interaction, leads to a detectable luminescent signal. (D) Quantitative analysis of the bioluminescence arising from the LgBiT-KRAS and HNB-HiBiT interaction in HEK293T cells, with non-fused HiBiT and LgBiT KRAS serving as control. Statistical significance of the interaction is denoted (p-values obtained by unpaired t-test).

(which was not certified by peer review) is the author/funder, who has granted bioRxiv a license to display the preprint in perpetuity. It is m
available under aCC-BY 4.0 International license.

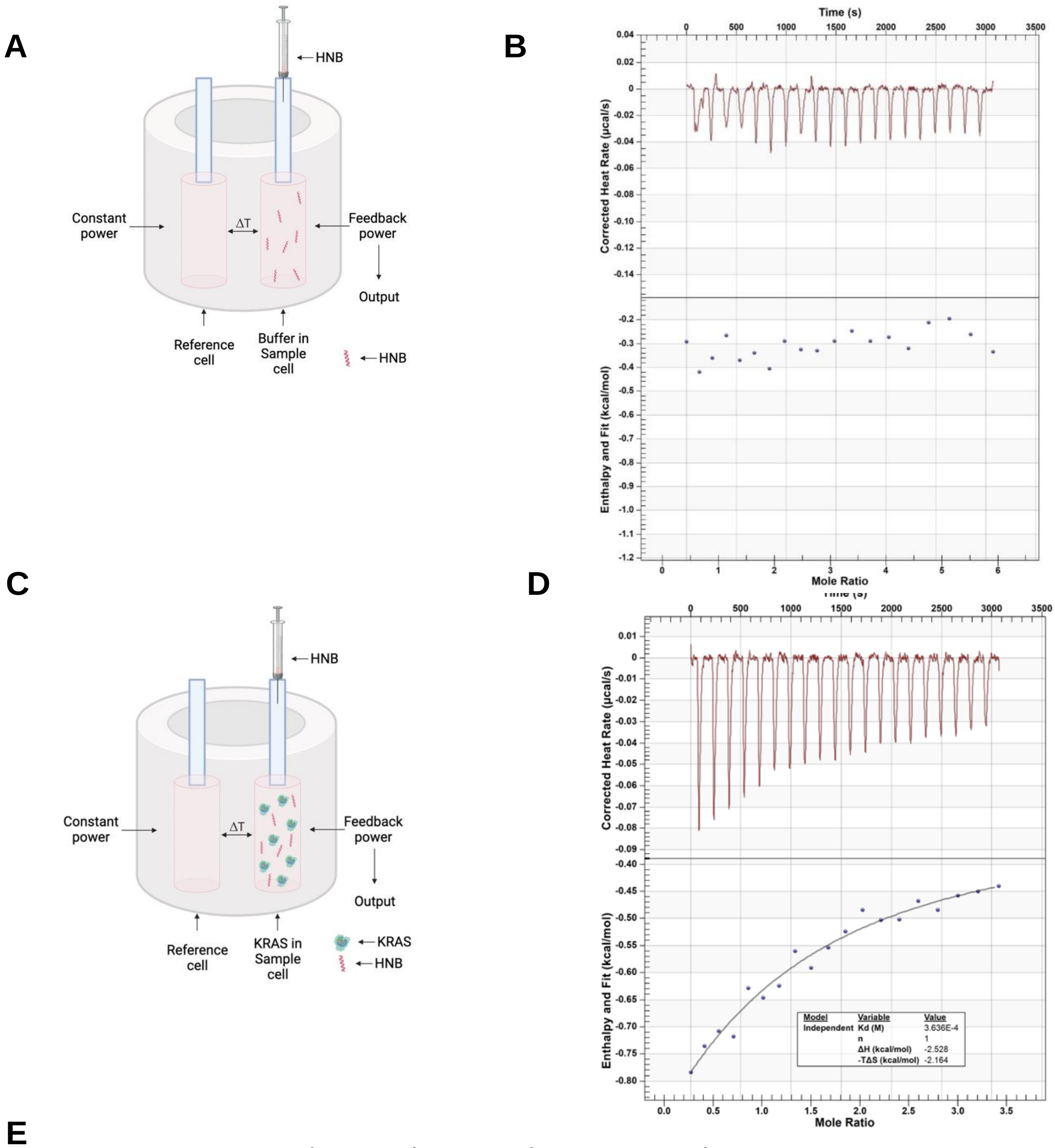

E

	K_d (μM)	N	DH (kcal/mol)	-TDS (kcal/mol)
HNB -> KRAS	370 ± 6	1 (fixed)	-4 ± 2	-0.7 ± 1.5

Supplementary Figure 21: Analysis of KRAS and HNB Interaction: (A) Schematic representation of an isothermal calorimeter used for HNB titration into ITC assay buffer, detailing the injection syringe filled with HNB and the sample cell containing the ITC assay buffer. (B) Graphical display of the titration of 500 μM HNB into the ITC assay buffer, serving as a baseline control, showing the thermal response over time. (C) Schematic representation of isothermal titration calorimeter with titration of HNB with recombinant KRAS, (D,E) The plot obtained from titrating 500 μM of HNB with 93 μM recombinant KRAS in ITC buffer showing dissociation constant (Kd) of 370 ± 6 μM. Analysis was done using the NanoAnalyze software

(which was not certified by peer review) is the author/funder, who has granted bioRxiv a license to display the preprint in perpetuity. It is m
available under aCC-BY 4.0 International license.

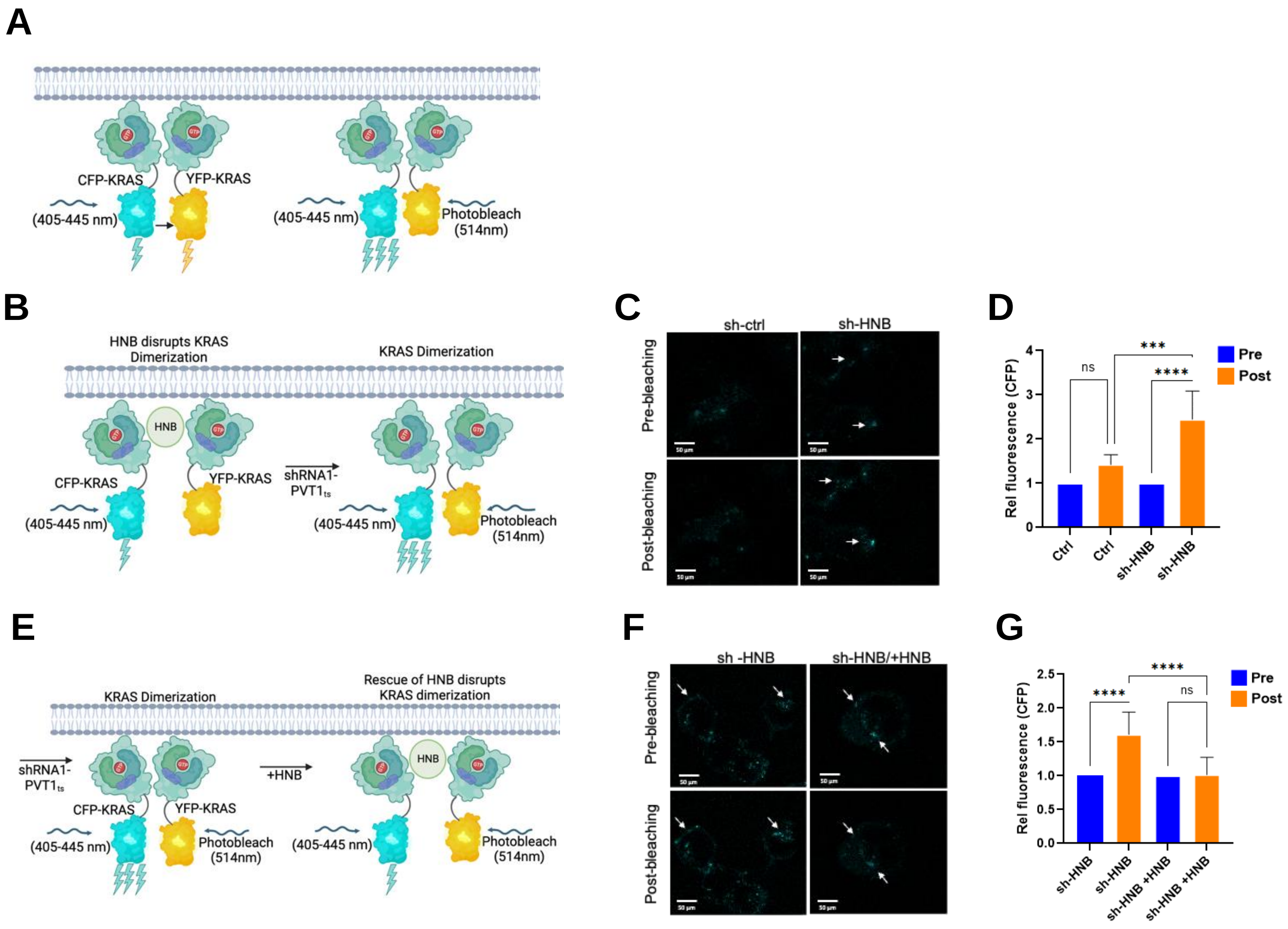

Supplementary Figure 22: HNB regulates KRAS dimerization (A) Schematic representation of KRAS dimerization using acceptor photobleaching FRET assay. (B) Schematic representation of KRAS dimerization on knocking down HNB using shRNA1-$PVT1_{ts}$. (C) Representative images showing signals for CFP-KRAS and YFP-KRAS. D458 sh.Ctrl and shRNA1.$PVT1_{ts}$ cells were co-transfected with CFP-KRAS and YFP-KRAS. Images were taken at 60X oil; the scale bar is 10µm. (D) CFP emission for KRAS in sh.Ctrl and shRNA1.$PVT1_{ts}$ D458 cells before (Pre) and after (Post) photobleaching of acceptor. (p-value calculated by one-way ANOVA, n=10). (E) Schematic representation showing disruption of KRAS dimerization upon expression of HNB using doxycycline induction. (F) Representative images showing signals for CFP-KRAS and YFP-KRAS. D458 shRNA1.$PVT1_{ts}$ and doxycycline-induced HNB-expressed D458 cells were co-transfected with CFP-KRAS and YFP-KRAS. Images were taken at 60X oil; the scale bar is 10µm. (G) CFP emission for KRAS in shRNA1.$PVT1_{ts}$ and +HNB in D458 cells before (Pre) and after (Post) acceptor photobleaching. (p-value calculated by one-way ANOVA, n=10).

(which was not certified by peer review) is the author/funder, who has granted bioRxiv a license to display the preprint in perpetuity. It is m
available under aCC-BY 4.0 International license.

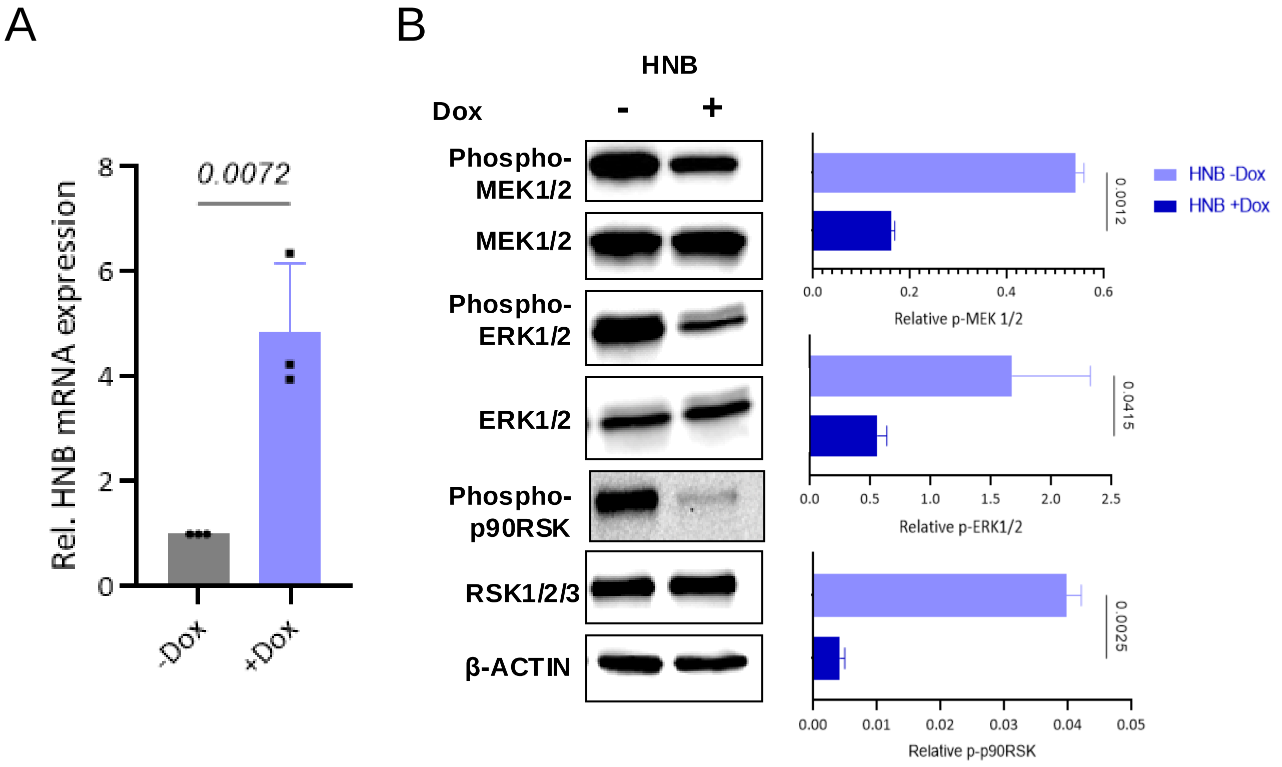

Supplementary Figure 23: Regulation of MAPK Signaling by HNB in NCI-H1792 Cells: (A) q-RT-PCR analysis showing quantification of HNB transcript levels in NCI-H1792 cells with (+Dox) or without induction (-Dox). (B) Western blot and quantitative analysis representing the expression levels of key signaling molecules: phospho-MEK1/2, total MEK1/2, phospho-ERK1/2, total ERK1/2, phospho-p90RSK, and total RSK1-2 in NCI-H1792 cells transduced with an inducible HNB-expressing plasmid. β ACTIN is utilized as a loading control (p-values obtained by unpaired t-test).

(which was not certified by peer review) is the author/funder, who has granted bioRxiv a license to display the preprint in perpetuity. It is m
available under aCC-BY 4.0 International license.

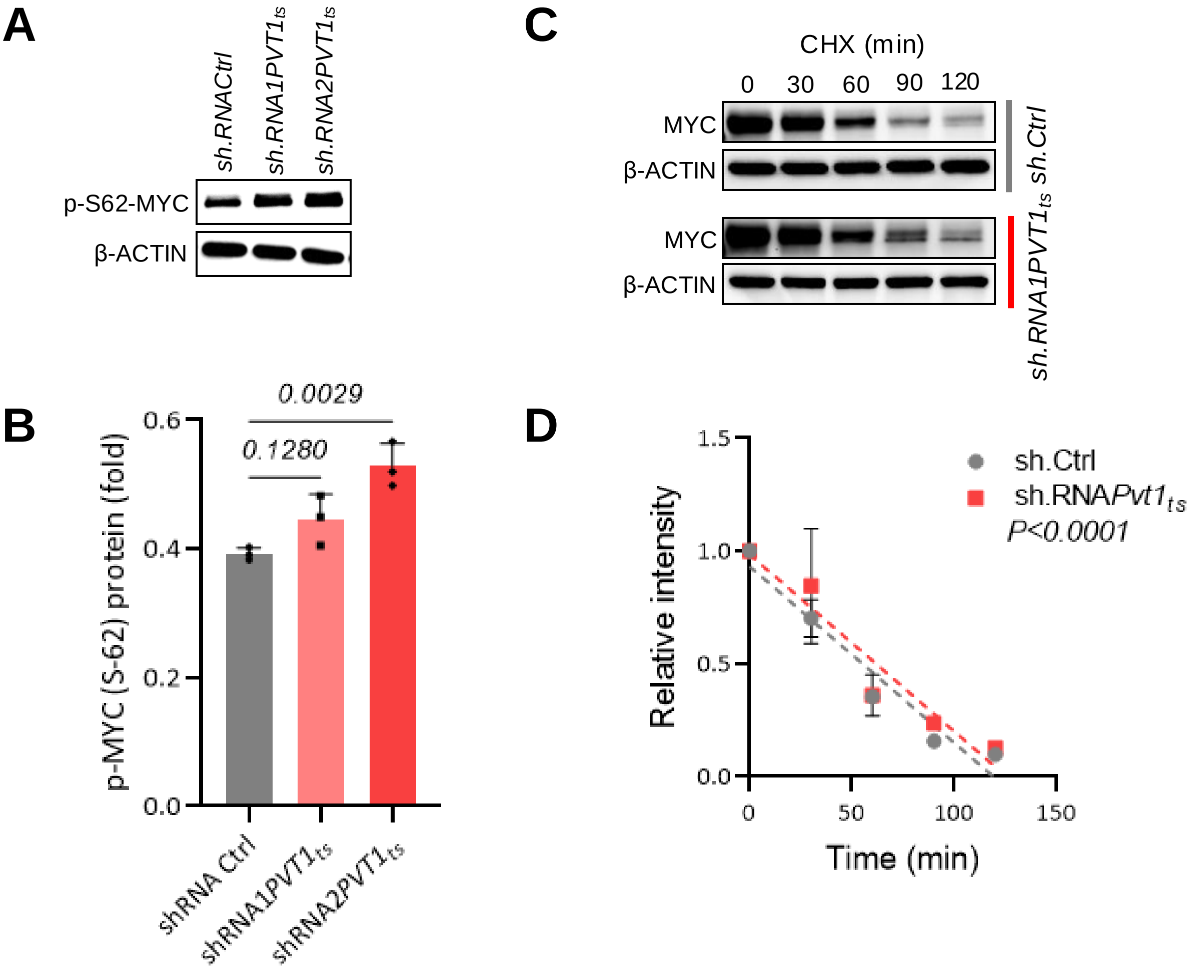

Supplementary Figure 24: Loss of HNB enhances MYC stability in NCI-H1792 **(A,B)** Western blot and quantitative analysis of p-MYC^{Ser62} in NCI-H1792 stably transduced with sh.Cntrl, shRNA1-PVT1$_{ts}$ and shRNA2-PVT1$_{ts}$. β ACTIN was used as loading control ((p-values obtained by ANOVA) . **(**C,D) Cycloheximide chase assay and quantification for MYC in NCI-H1792 cells transduced with sh.Cntrl (as control) or shRNA1-PVT1ts. β-ACTIN was used as a loading control. (P value obtained by simple linear regression)
